# Open pangenome of *Lactococcus lactis* generated by a combination of metagenome-assembled genomes and isolate genomes

**DOI:** 10.3389/fmicb.2022.948138

**Published:** 2022-08-23

**Authors:** Yiting Zhai, Chaochun Wei

**Affiliations:** Department of Bioinformatics and Biostatistics, School of Life Sciences and Biotechnology, Shanghai Jiao Tong University, Shanghai, China

**Keywords:** *Lactococcus lactis*, fermented food, pangenome, metagenome, metagenome-assembled genomes (MAGs)

## Abstract

*Lactococcus lactis* (*L. lactis*) is a well isolated and cultured lactic acid bacterium, but if utilizing the isolate genomes alone, the genome-based analysis of this taxon would be incomplete, because there are still uncultured strains in some ecological niches. In this study, we recovered 93 high-quality metagenome-assembled genomes (MAGs) of *L. lactis* from food and human gut metagenomes with a culture-independent method. We then constructed a unified genome catalog of *L. lactis* by integrating these MAGs with 70 publicly available isolated genomes. Having this comprehensive resource, we assessed the genomic diversity and phylogenetic relationships to further explore the genetic and functional properties of *L. lactis*. An open pangenome of *L. lactis* was generated using our genome catalog, consisting of 13,066 genes in total, from which 5,448 genes were not identified in the isolate genomes. The core genome-based phylogenetic analysis showed that *L. lactis* strains we collected were separated into two main subclades corresponding to two subspecies, with some uncultured phylogenetic lineages discovered. The species disparity was also indicated in PCA analysis based on accessory genes of our pangenome. These various analyzes shed further light on unexpectedly high diversity within the taxon at both genome and gene levels and gave clues about its population structure and evolution. *Lactococcus lactis* has a long history of safe use in food fermentations and is considered as one of the important probiotic microorganisms. Obtaining the complete genetic information of *L. lactis* is important to the food and health industry. However, it can naturally inhabit many environments other than dairy products, including drain water and human gut samples. Here we presented an open pan-genome of *L. lactis* constructed from 163 high-quality genomes obtained from various environments, including MAGs recovered from environmental metagenomes and isolate genomes. This study expanded the genetic information of *L. lactis* about one third, including more than 5,000 novel genes found in uncultured strains. This more complete gene repertoire of *L. lactis* is crucial to further understanding the genetic and functional properties. These properties may be harnessed to impart additional value to dairy fermentation or other industries.

## Introduction

*Lactococcus lactis* (*L. lactis*) is a Gram-positive, catalase-negative, mesophilic, facultatively anaerobic microorganism performing homolactic fermentation (ca. 95% of glucose is converted to lactate) (Neves et al., 2005). It is divided into four subspecies: *lactis*, *cremoris*, *hordniae* (leaf hopper), and *tructae* (trout intestine) (Perez et al., 2011). Based on the long history of safe use in food fermentation, its application in food is generally regarded as safe (GRAS) (Food and Drug Administration [FDA], 2010). It is one of the major lactic acid bacteria (LAB) used worldwide for the production of numerous dairy products including cheese, fermented milk (Mataragas, 2020), and other fermented foods such as kimchi (Jung et al., 2011) and sausages (Dowdell et al., 2020). Although *L. lactis* is most widely known for its association with the milk environment, it can naturally inhabit many other environments. Strains of this species have been isolated from a range of sources including drain water (Kato et al., 2012) and human vaginal (Todorov et al., 2007; Gao et al., 2011) samples. It can also survive in the human gastrointestinal tract, which in turn has opened up the potential of this species for probiotic use, production of recombinant proteins, and the delivery of therapeutic drugs *in-vivo* (Mataragas, 2020). The ability of this taxon to colonize a larger ecological niche is associated with greater genomic diversity (Passerini et al., 2010).

Lactic acid bacteria present a high degree of 16S rRNA gene sequence similarity (Mataragas, 2020). For example, the two subspecies, *L. lactis* subspecies (subsp.) *lactis* and *L. lactis* subsp. *cremoris*, differ by less than 0.7% in their 16S rDNA sequences but display an average of only 85% DNA identity at the genome level (Passerini et al., 2010). Consequently, although the 16S rRNA gene is widely used for bacterium identification purposes, it is not suitable for distinguishing *L. lactis* subsp. *lactis* from *L. lactis* subsp. *cremoris*. Using whole genomes of the microorganisms to investigate the genetic diversity and functional features of *L. lactis* is more appropriate (Chun et al., 2017). The advance in modern sequencing technologies has made whole-genome sequences more accessible, and as a result, there are now 293 lactococcal assemblies publicly available in the NCBI (National Center for Biotechnology Information) database (as of March 5th, 2022), 70 of which are at complete- or chromosome-level assemblies (45 genomes belonging to *L. lactis* subsp. *lactis* and 25 genomes belonging to *L. lactis* subsp. *cremoris*). A large number of genome sequences available facilitates the exploration of the genetic, metabolic, and physiological properties of the isolated strains (Cavanagh et al., 2015b). This makes it possible to gain an understanding of the genomic and genetic diversification of the taxon in great detail.

To provide a holistic insight of *L. lactis* from different perspectives like genomic characteristics, genetic diversity, and metabolic properties, Mataragas studied the phylogenetic relationship, genetic properties, and metabolic capabilities of the *L. lactis* subsp. *lactis* strains using the isolate genomes available in the GenBank database (Mataragas, 2020). The chromosomal features of 30 *L. lactis* strains were assessed with particular emphasis on discerning the subspecies division, evolution, and niche adaptation by Kelleher (Kelleher et al., 2017). Clonal diversification and phenotypic variability of *L. lactis* subsp. *lactis* strains essentially arose through substantial genomic flux within the accessory genome (Passerini et al., 2010). However, these studies only utilized the genomes of strains that had been isolated and cultured, they gave little information about the strains in the original state because some strains are yet not cultured. Metagenome sequencing, on the other hand, provides a culture-independent method to capture the entire DNA content of an environment, which can help us understand the complete genetic information in the real environment, including genes of uncultured strains. Some other studies have built genome catalogs using metagenome-assembled genomes (MAGs) and isolated genomes before (Almeida et al., 2021; Kim et al., 2021; Nayfach et al., 2021), which only focused on the whole earth microbiome or gut microbiome. A definite unmet demand exists for genome catalogs of a particular species such as various lactic acid bacteria valuable in practical applications.

In our study, for obtaining more comprehensive genetic information on *L. lactis*, we not only used the isolate genomes but also added the MAGs recovered from metagenomes of different environments. To our knowledge, although there was exploration to combine MAGs and isolate genomes from the same sample to improve the understanding about the fecal samples (Meziti et al., 2021), our study focused on in-depth pan-genomic analysis for a species, *L. lactis*, combining MAGs and cultivated genomes. The development of such a curated catalog is crucial to further expand gene pool. In addition, the increased dataset of lactococcal genomic sequences allows for deeper analysis like pangenome analysis, Gene Ontology (GO) analysis, phylogenetic analysis, average nucleotide identity (ANI) calculation, and so on with various bioinformatics tools. We shed further light on the diversity within the *L. lactis* species and identified more genes present in these strains. This genomic or genetic diversity could provide a new perspective to identify novel starter cultures with the desired industrial traits for production to develop products with improved quality and sensory attributes (Mataragas, 2020).

## Results

### Genome collection of *Lactococcus lactis* existing in food and human gut metagenomes

**FIGURE 1:**
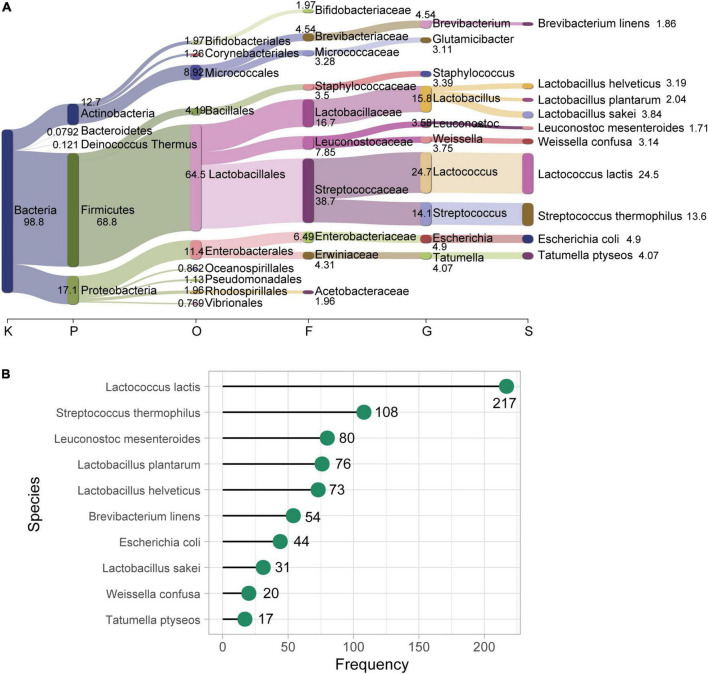
Bacterial species in food metagenomes. **(A)** Taxonomic profile of food microbiome at different taxonomic levels. The top ten most abundant species at different taxonomic levels (Kingdom, Phylum, Order, Family, Genus, and Species) are displayed. The thickness of the flow (and the number beside the boxes) represents the average relative abundance (%) of each taxon, which is the average of relative abundance of all 323 food samples. Visualized with Pavian (Breitwieser and Salzberg, 2020). **(B)** The prevalence of the ten most abundant species in our food samples. The x-axis represents the frequency of positive samples for each species (y-axis) in the entire 323 food samples.

We collected 323 food metagenomes, which corresponded to different types of fermented foods (15 datasets of food samples; Table 1). In addition, we considered 1,149 human gut metagenomes from several projects (Table 1) (see Materials and methods for more details). Taxonomic analysis of the food metagenomes revealed that these communities contained 638 bacterial species in total (Supplementary Table 1). Many species commonly found in fermented foods, such as *Lactococcus lactis*, *Streptococcus thermophilus*, *Lactobacillus helveticus*, and *Lactobacillus plantarum*, as well as some conditional pathogens, such as *Escherichia coli*, were identified in food metagenomes. *Lactococcus lactis* was the most abundant species in our food samples with an average relative abundance of about 25% at the species level considering all 323 food samples (Figure 1A) and 37.1% when only considering *L. lactis* positive samples. We also counted the frequency of occurrence of this taxon, detecting 217 (a proportion of 67.2%) samples were positive for it (Figure 1B). The second most abundant and prevalent species in our food samples was *Streptococcus thermophilus* which was also a member of LAB, with an average relative abundance of 13.6% and prevalence of 33.4% (108 out of 323). However, not like the widespread distribution in food samples, the average relative abundance of *L. lactis* is far lower (1.97% only considering positive samples), and the occurrence of *L. lactis* in human gut samples was much less, only 106 out of 1,149.

**TABLE 1:** Summary of the collected food and human gut metagenomic datasets.

Project accession	Sample source	Sample size	Database	Date of data release
PRJNA603575	Yogurt and dietary supplement	16	NCBI	2020/4/24
PRJNA185981	Soy sauce	7	NCBI	2013/3/16
PRJNA730347	Soy sauce	6	NCBI	2021/5/20
PRJEB6952	Cheese	10	NCBI	2016/4/2
PRJEB15423	Cheese	42	NCBI	2017/12/5
PRJNA430402	Cheese	36	NCBI	2018/1/26
PRJEB32768	Cheese	77	NCBI	2019/11/30
PRJEB6314	Cheese	5	NCBI	2015/1/29
PRJNA482503	Cheese	6	NCBI	2018/11/10
PRJNA286900	Cheese	1	NCBI	2016/2/24
mgp3362	Cheese	24	MG-RAST	2013/6/2
PRJEB35321	Fermented food	58	NCBI	2020/10/27
PRJNA603605	Indonesian food	3	NCBI	2020/1/30
PRJNA352236	Sausages	16	NCBI	2016/11/6
PRJNA305659	Wine	16	NCBI	2016/12/15
**No. of food samples**		**323**		
PRJEB9584	Human gut	211	NCBI	2017/8/2
PRJNA231909	Human gut	542	NCBI	2015/4/18
PRJNA422434	Human gut	370	NCBI	2015/2/6
Pasolli’s collection	Human gut	26	https://opendata.lifebit.ai/table/sgb	/
**No. of human samples**		**1,149**		

**FIGURE 2:**
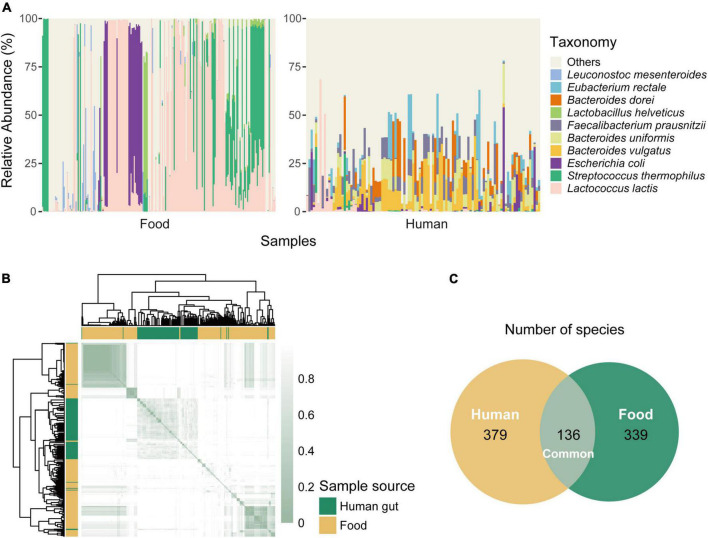
Differences in species composition between food and human gut samples. **(A)** Species composition of food and human gut metagenomes. We only select samples that were positive for *Lactococcus lactis* and then display the ten most abundant species among all samples. The left part shows the relative abundance of each food sample, while the right part is for gut samples. **(B)** Difference of species abundance between samples. Samples positive for *L. lactis* are used and Bray-curtis distances are calculated. The smaller the value of the distance is (the deeper the color is), the more similar the two samples are. Annotation strip distinguishes the sample sources, green marks human samples, and yellow marks food samples. **(C)** The number of species present in food and human niches. There are 136 common species, 379 species unique to human samples, and 339 species unique to food samples.

Although not an endogenous inhabitant of the gastrointestinal tract, *L. lactis* was capable of surviving in the gut passage (Cavanagh et al., 2015b). From the relative abundance profiles, we knew *L. lactis* existed in both food and the human gut, although the abundance of this taxon in the two niches was different. We next analyzed the difference in community composition and microbial diversity between the two environments. All samples were screened for *L. lactis* and selected positive samples for downstream taxonomic analysis. The variance of relative abundance of *L. lactis* in all human samples was 0.0071, while that in food samples was 0.1604. It suggested that the content of *L. lactis* among human samples was more stable probably because of the widespread low abundance. Food and human gut samples that we picked out contained at least one same species (*L. lactis*), but species composition structure had obvious differences (Figure 2A). Dominant species present in *L. lactis* positive human metagenomes were *Bacteroides vulgatus*, *Bacteroides uniformis*, *Faecalibacterium prausnitzii*, and *Bacteroides dorei*, while *Lactococcus lactis* and *Streptococcus thermophilus* were predominant in food positive samples.

### Comparison of community composition structure (or organism composition) in food and human gut metagenomes

To quantize the difference, we computed the Bray-Curtis distance indices which represented the similarity between the two community structures (or bacterial composition) derived from the two metagenomes. The smaller the distance, the more similar the two samples were in terms of community structure (or organism composition). It was clear that food and gut samples were clustered separately (Figure 2B), meaning that the bacterial composition similarity between food and gut environments was limited, and this suggested that each group had its distinct microbial diversity. We also counted the number of species existing in each niche. There were 136 common species, while the human niche contained more distinct species than the food niche, 379 unique species for human samples and 339 for food samples (Figure 2C). These differences suggested that the two niches had different community structures, resulting in the different species’ competitive patterns and bacterial interactions, which would influence the genetic and functional characteristics of *L. lactis* to better adaption and fitness.

### Construction of a high-quality genome catalog of *Lactococcus lactis* from food and human gut metagenomes and isolated genomes

**FIGURE 3:**
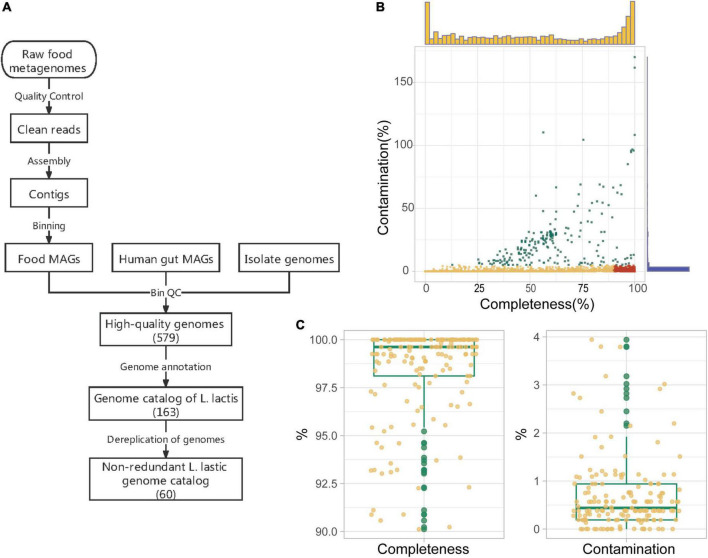
Construction and quality assessment of genome catalog *Lactococcus lactis*. **(A)** The pipeline for the construction of *L. lactis* genome catalog. Reads quality control, assembly, and binning were performed on food metagenomes to gain food microbiota metagenome-assembled genomes (MAGs). Then we carried out genome quality assessment over all genomes (including the human gut and food microbiota MAGs and isolate genomes). All high-quality genomes (Completeness > 90% and contamination < 5%) were incorporated together for genome annotation. In the end, there were 163 genomes belonging to *Lactococcus lactis*, and the number of non-redundant genomes was 60. **(B)** Completeness and contamination values of 2,086 food metagenomic bins. High-quality, medium-quality, and low-quality food microbiota MAGs were marked in red, yellow, and green, respectively. Marginal histograms added on the X and Y-axis represent frequencies of bins corresponding to different completeness (yellow) /contamination (blue) values. **(C)** Quality of 163 *L. lactis* genomes in our genome catalog. Box lengths represent the IQR of the data, and the whiskers represent the lowest and highest values within 1.5 times the IQR from the first and third quartiles, respectively.

We obtained genomes of *Lactococcus lactis* from both isolated genomes and metagenomes and then constructed a unified genome catalog (Figure 3A). There are three main sources of genomes: food microbiota MAGs, human gut microbiota MAGs, and isolate genomes. Strains of isolated *L. lactis* genomes were mainly isolated from dairy products, or fermented plant materials, and some isolates were from human, meat, and sink drain water. For the raw sequence data of food metagenomes, we first filtered and trimmed human genome sequences and contaminant sequences. After getting clean sequencing data, we performed single-metagenome assembly and contig binning. Finally, we gained 2,086 food metagenomic bins in total. Assessment of genome quality showed that 894 food microbiota MAGs satisfied the medium-quality criteria (>50% completeness and < 5% contamination), of which 497 food microbiota MAGs were of high-quality (>90% completeness and < 5% contamination) (Figure 3B). In addition, human gut microbiota MAGs annotated as *L. lactis* and isolate genomes were directly downloaded from public databases. Then quality control was also conducted. Only 12 out of 29 human microbiota MAGs passed high-quality criteria, and all 70 isolate genomes passed. All of these high-quality genomes (MAGs and isolate genomes) were integrated into a collection of qualified genomes (579 genomes) that was used for the following analysis.

We next performed genome taxonomic annotation on all 579 qualified genomes using Phylophlan and GTDB-Tk. Eighty-one food microbiota MAGs were classified as *L. lactis* (10 *L. lactis* subsp. *cremoris* and 71 *L. lactis* subsp. *lactis*), and all 12 human microbiota MAGs (all belonging to *L. lactis* subsp. *lactis*) and 70 isolate genomes (25 *L. lactis* subsp. *cremoris* and 45 *L. lactis* subsp. *lactis*) we collected were also verified as this taxon. A total of 163 genomes made up the genome catalog of *L. lactis* and their quality information about completeness and contamination was shown (Figure 3C). This general genome catalog of *L. lactis* integrated genomes of multiple sources, that is, isolate genome, food microbiota MAGs, and human gut microbiota MAGs, containing more genomic and genetic information.

### Non-redundant genomes of *Lactococcus lactis* reveal some uncultured lineages

**FIGURE 4:**
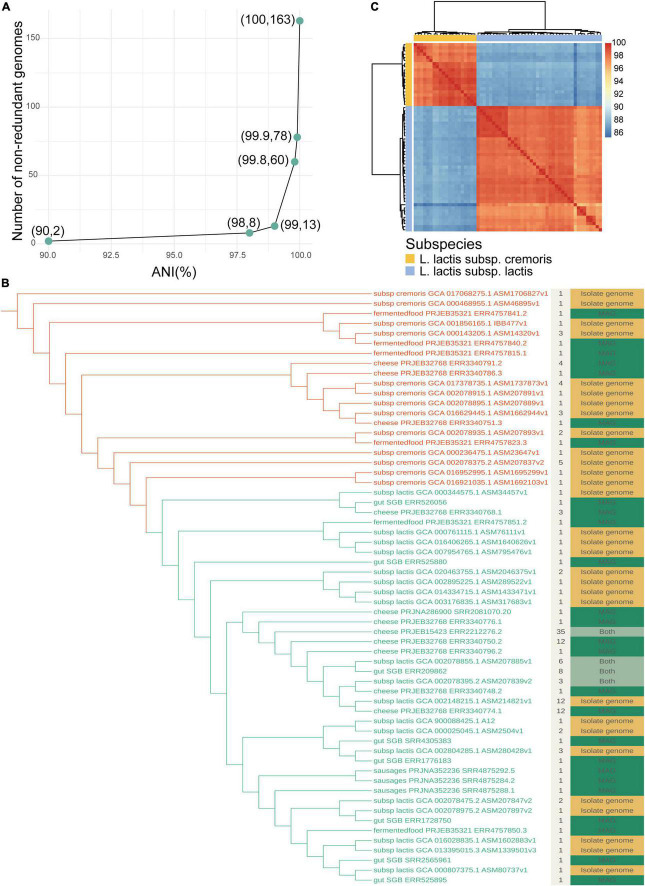
Intraspecies diversity of *Lactococcus lactis*. **(A)** The number of non-redundant genomes with different ANI. ANIs of 90%, 98%, 99%, 99.8%, 99.9%, and 100% were used to remove redundancy. **(B)** Maximum-likelihood phylogenetic tree of non-redundant genomes belonging to *L. lactis*. The tree showing all 60 dereplicated genomes was computed using a core gene alignment file generated from pangenome analysis, bootstrapped *1000 replicates. The colors of branches distinguish subclades: *L. lactis* subsp. *cremoris* (marked in red) and *L. lactis* subsp. *lactis* (marked in green). The layer beside the tree displays the number of genomes included in each clustering group. The outmost layer is colored according to whether the strain of each group has been cultured, where green represents that all genomes are in a culturable state, yellow represents that all genomes are uncultured and the light green represents that both states are present. **(C)** Similarity heatmap of all non-redundant genomes. Each row (and column) represents a genome and the fill value shows the ANI between each pair. The yellow strip marks genomes belonging to *L. lactis* subsp. *cremoris* and blue marked *L. lactis* subsp. *lactis.*

For exploring intraspecies diversity, we assessed the similarities between genomes belonging to *L. lactis* by calculating the average nucleotide identity (ANI). We then removed redundancy by selecting a single representative for each clustering of genomes that shared an ANI of greater than 99.8% which was also used as a threshold value in a previous study (Shaiber et al., 2020). This generated a final collection of 60 non-redundant genomes, of which 20 belonged to *L. lactis* subsp. *cremoris* and 40 belonged to *L. lactis* subsp. *lactis* (Figure 4B). If we changed the value of ANI to 99.9% which was used by Kim (Kim et al., 2021) and Almeida (Almeida et al., 2021), 78 non-redundant genomes were generated (Figure 4A). The core genome-based phylogeny substantiated the early separation of the *L. lactis* subspecies *lactis* and *cremoris*. There were 35 genomes clustered into the largest group ‘cheese_PRJEB15423_ERR2212276.2,’ which represented a strain common in both cultured and culture-independent states. We found that 26 (43.3%) of the clustering groups did not have a genome in NCBI cultured database, i.e., only included MAGs, which may be due to the incompleteness of the current database or identifying uncultured strains. The non-redundant catalog expanded the known phylogenetic diversity of *L. lactis* by about 43%. For the four groups containing both isolate genomes and MAGs, two of these groups were represented by MAGs (group ‘cheese_PRJEB15423_ERR2212276.2’ and group ‘gut_SGB_ERR209862’), suggesting that reconstructed genomes were of better quality than isolate genomes downloaded in publicly available databases.

With the development of sequencing and computational technologies, the combination of core-genome phylogeny and average nucleotide identity (ANI) values could provide accurate taxonomic guidance based on whole-genome sequences (Xu, 2006). Pairwise similarities for all genomes using ANI showed that the sequence identities between the two subspecies were low with ANI of less than 90% (Figure 4C). The lowest value for the *L. lactis* species was 85.2%, while the highest ANI value was 99.9%, revealing that the genomic sequence identities between different subclades had marked differences. ANI could be used as a reference characteristic for the identification of *L. lactis* subspecies. Phylogenetic analysis provided knowledge of intraspecies diversity and new lineages, and ANI further estimated the similarity between strains.

### Pangenome analysis combining metagenome-assembled genomes (MAGs) and isolate genomes reveals more genetic diversity

Phylogenomic analysis inferred ancestral relationships by the evolution of core genes. Yet, phylogeny as predicted by core genes may not uniformly explain the distribution of accessory genes and singletons across genomes, including genes that may be critical determinants of fitness against particular selective environmental pressures (Dutilh et al., 2004). In contrast to phylogenomic analysis, pangenome analysis revealed associations between genomes based on the presence or absence of all accessory genes across genomes. Pangenome can more effectively capture ecological dissimilarities between genomes due to the strong influence of accessory genes (Delmont and Eren, 2018). To evaluate current sequencing efforts of the *L. lactis* and to explore if the genomes recovered from environmental metagenomes could provide a more comprehensive overview of the genetic diversity, pangenome analysis was performed on only isolate genomes and all 163 genomes (isolate genomes and MAGs), respectively. Prokka (Seemann, 2014) was used to annotate the genomes. The resulting .gff files were subjected to pangenome analyzes using Roary (Page et al., 2015) with a minimum amino acid identity for a positive match at 80%. Genes present in more than 95% of genomes were defined as the core genes, while those only existing in a single strain were classified into singletons. The remaining genes were defined as accessory genes. The presence and absence of non-singleton genes (i.e., occurred in at least 2 genomes) across 163 genomes were shown in the circular heatmap (Supplementary Figure 1). We used hierarchical clustering to group together the genes that showed similar distribution patterns across genomes.

**FIGURE 5:**
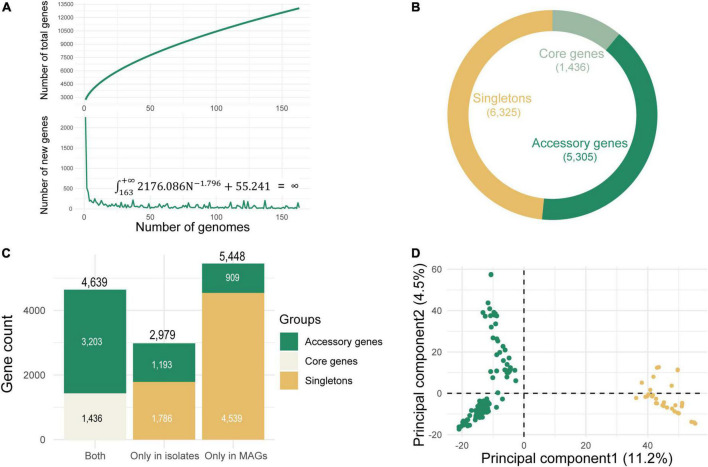
Pangenome analysis of *Lactococcus lactis* using all 163 conspecific genomes. **(A)** Pangenome size estimation. The growth curve represents the number of total genes in the pangenome, and the decreasing curve represents the number of new genes. Estimating the size of new genes based on a power function (*n* = kN*^r^*+a), where n represents the number of additional new genes detected for each additional genome sample, N represents the number of genome samples, and k, r and a are three parameters of this model. The points used for fitting are the mean of 10 random sampling. The fitting result is *k* = 2176.086 (2082.090, 2270.150); *r* = −1.796 (−1.980, −1.641); *a* = 55.241 (47.308, 63.108) (R^2^ = 0.935), and the integral is infinite: $\int_{163}^{+\infty }2176.086\,{\text{N}}^{-1.796}+55.241$ = ∞. The values in brackets are 95% confidence intervals. As for fitting of total number of genes, the deduced mathematical function is y = 627.412x^0.561^+2105.194, R^2^ = 0.998. With the increasing number of genomes, the size of the pangenome continues to grow and does not reach a plateau stage, indicating an open pangenome. **(B)** The number of core, accessory, and singleton genes in pangenome. **(C)** The number of genes unique to metagenome-assembled genomes (MAGs), unique to isolate genomes, and common in two groups. **(D)** Separation of the subspecies of *L. lactis* subsp. *lactis* and *L. lactis* subsp. *cremoris* based on accessory genes (*n* = 5,305) detected in the Roary-generated open pangenome.

For closed pangenomes, adding new genomes will not lead to the discovery of novel genes, whereas for open pangenomes, each new genome sequence usually reveals new members of the gene pool for that species (Bosi et al., 2015). The approach for estimating the pangenome size has been pioneered by Tettelin et al. (2005). The value of the novel gene discovery rate is used for extrapolating the pangenome size, which will be asymptotically stabilized at a certain value. For an open pangenome, this value is nonzero, and the pangenome size cannot be estimated, (i.e., its integral is infinite). The resulting graph revealed an asymptotic curve increasing of pangenome size without reaching a plateau, and the integral of the fitted curve of novel gene size was infinite (Figure 5A). It suggested that the pangenome generated by 163 conspecific genomes was in an open state. This plenty of room for the growth of novel genes suggested that even for common, well-studied *L. lactis*, a surprising amount of intraspecies genetic diversity remained to be sequenced and captured. A total of 13,066 genes were detected in the open pangenome: 1,436 of them belonged to core genes; the number of accessory genes was 5,305 and the remaining 6,325 genes were strain-specific genes (singletons) (Figure 5B). We observed the number of singletons was almost equal to that of non-singletons. This suggested that genetic diversity was largely the consequence of substantial gene diversification within the singleton genes. MAGs constituted the major contributors to the genetic diversity observed within the species. The number of genes only existing in isolate genomes was 2,979, while there were more genes (5,448) unique to MAGs (Figure 5C). The difference in gene content between MAGs and isolates was mainly due to singletons because the number of singletons in MAGs was more than twice than that in isolate genomes. The metagenomically reconstructed genomes greatly expanded the genetic diversity of *L. lactis*. These expanded genome sets provided much larger collections of distinct genes that were present in various strains. PCA analysis based on the accessory genes clustered genomes into two different subclades (Figure 5D): groups of *L. lactis* subsp. *lactis* and groups of *L. lactis* subsp. *cremoris*, which agreed with our phylogenetic analysis of the core genes and was consistent with the NCBI taxonomy of isolate genomes. The Pangenome of *L. lactis* generated by only isolate genomes was also in an open state (Supplementary Figure 2). When considering MAGs and isolate genomes simultaneously, the pangenome grew at a much higher rate. The overall genome sets described a more real and complete genetic repertoire of this taxon indicating that there was so much potential for expansion.

The genes of our pangenome were also annotated with a rich set of functional descriptions. After considering MAGs, pangenome contained more genes, with a substantially wider functional potential. Overall, 9,442 genes (72.3% of all genes) were assigned at least a functional annotation based on EggNOG orthology data (Huerta-Cepas et al., 2016), of which 751 were assigned GO (Gene Ontology) labels. This rich gene annotation of the pangenome enabled a comprehensive functional characterization of *L. lactis*.

To distinguish the functions encoded in the core, accessory and singleton genes, we performed functional enrichment analysis of the selected gene set based on the GO functional categories. Genes classified as core were significantly associated (*p*-value < 0.05; *q*-value < 0.05) with key metabolic functions involved in amino acid metabolism, nitrogen compound metabolism, organic cyclic compound metabolism and aromatic compound metabolism, as well as other housekeeping functions (e.g., intracellular anatomical structure, catalytic activity, RNA modification and ligase activity) (Supplementary Figure 3). This observation was typical since genes involved in such processes were required for the maintenance of basic cellular function and were expressed in nearly all microbial cells (Mataragas, 2020). Besides, we identified some functions associated with the regulation of transcription and viral activities significantly enriched (*p*-value < 0.05; *q*-value < 0.05) in accessory genomes (Supplementary Figure 4), which included genes identified only in parts of the genomes. Genes involved in processes like DNA and protein binding, membrane-bounded organelle and response to chemicals were predominantly present in a single genome (Supplementary Figure 5). In addition, the large number of *L. lactis* genomes we recovered from environments allowed us to investigate functions of the genes shared by reconstructed genomes by examining the functions encoded by genes only existing in MAGs. These enriched functions primarily included the protein binding, metabolic and biosynthetic process of lipid, polysaccharide and lipopolysaccharide (Supplementary Figure 6). The organization of the reconstructed genomes and their functional profiling would be the basis for comprehensive metagenomic characterizations.

## Discussion

*Lactococcus lactis* is a well isolated and cultured bacterium, but there could still be some novel strains or genes undiscovered in real ecological niches (Laroute et al., 2017). In this study, we recovered 81 food microbiota MAGs and collected 12 human microbiota MAGs other than isolate genomes. Then we integrated these MAGs with 70 publicly available isolate genomes to construct a general genome database of *L. lactis*. For further understanding of the characterization of *L. lactis*, we then assessed the genomic characteristics, phylogenetic relationships, and genetic diversity of a collection of 163 genomes. The various analysis revealed unexpectedly high diversity within the taxon at both genome and gene levels and gave clues about its population structure and evolution. This has shed further light on the diversity within the *L. lactis* species and identified genes present in these strains. The diversity will enable rational selection of optimized candidates not only for dairy products but also for non-food applications, including white biotechnology or health issues.

Before reconstructing genomes from metagenomes, we studied the presence or absence of *L. lactis* among the metagenomes we collected. Utilizing the species annotation results of food metagenomes, we detected a high prevalence and abundance of *L. lactis* in our food samples. This result was almost consistent with Pasolli and colleagues’ work (Pasolli et al., 2020) where *L. lactis* was the second prevalent species and *Streptococcus thermophilus* was the most prevalent one. The minor difference was probably a consequence of different datasets. The species composition was significantly different between food and human gut niches containing *L. lactis*. There were a large number of species only existing in a certain niche. The total number of species exclusively detected in food samples were 339, including some *Bifidobacteria*. This is consistent with the previous finding (Pasolli et al., 2020) that the majority of the species found in food samples did not overlap with the species found in human gut niches. *L. lactis* existing in different environments faced different bacterial interactions and competitions, which may contribute to different gene content related to niches adaption.

Metagenome-assembled genomes (MAGs) were used in many previous papers about the microbiome (Pasolli et al., 2019; Shaiber et al., 2020; Almeida et al., 2021), the application of genome-resolved metagenomics could provide new genomes for prevalent yet uncultivated members of the microbiome. Our study recovered 81 high-quality MAGs from food samples. The number of *L. lactis* positive samples in our study was larger than the previous study by Pasolli (Pasolli et al., 2020), therefore we recovered more high quality genomes (under the criteria of completeness > 90% and contamination < 5%). Some of these genomes can inform future cultivation efforts as they suggest the existence of novel lineages with no cultured representatives and can enrich future comparative genomics and metagenomic read recruitment studies as they increase the known microbial gene pool and diversity. By integrating publicly available isolate genomes and MAGs that may belong to the strain not been isolated and cultured, we could construct a more general genome catalog of *L. lactis*. This work expanded the collection of lactococcal genomes by more than doubling the current collections of 70 isolated genomes. We removed redundancy of all lactococcal genomes under the average nucleotide identity (ANI) of 99.8%, a standard that was also used in Shaiber’s work (Shaiber et al., 2020). Finally, we got sixty non-redundant genomes of *L. lactis*. We found that 26 clustering groups (43.3%) did not contain any isolate genomes, indicating that the large uncultured diversity remains within *L. lactis*. This unprecedented view of intraspecies diversity within *L. lactis* is far beyond the scope offered by current isolate genomes. Getting the number of conspecific genomes of *L. lactis* is beneficial to gaining knowledge of intraspecies diversity. In addition, the resulting high genome variability suggests a large pangenome for the species, which would be of great help in capturing more genetic and functional characteristics of the taxon.

The pangenome generated either by total genomes (isolate genomes and MAGs) or only by isolate genomes was in an open state, and the size of the former one increased at a higher rate. Their size increased indefinitely when adding new genomes, thus sequencing additional genomes would likely yield novel genes. The pangenome of *L. lactis* subsp. *lactis* produced by Mataragas (2020) was also open, and they generated a pangenome of 5,478 genes only using publicly available isolate genomes, with a threshold of sequence identity equal to 50%. MAGs constituted the major contributors to the genetic diversity observed within the species, with only 7,618 genes existing in isolate genomes while 10,087 in MAGs. Although we performed stringently quality control on raw reads and genomes we used, there might be some contamination from the process of data sequencing and processing to generate the MAGs, which may lead to an inflated pangenome size. Most of the genes in the pangenome (72.3%) were annotated with some functional modules like GO and KEGG, and function enrichment analysis gave clues about the key functions of each special gene set. The functional predictions generated for the pangenome could also be leveraged to develop new culturing strategies for the isolation of candidate strains. Moreover, our analysis distinguished genomes belonging to subspecies *lactis* and *cremoris* based on different genetic makeup. This demarcation was observed in the phylogenetic tree built using the core sequences and PCA analysis based on accessory genes, with two subgroups that corresponded to each subspecies. These observations supported the taxonomic classification of *L. lactis* by Cavanagh based on ANI (average nucleotide identity) and TETRA (tetranucleotide frequency correlation coefficients) (Cavanagh et al., 2015a) and by Kelleher based on carbohydrate and amino acid metabolism (Kelleher et al., 2017). Overall, a better understanding of lactococcal gene content and function could be of great importance and be an applicable tool in selecting special genes to be used in fermentation or other industries (Laroute et al., 2017).

The rapid growth curve of pangenome size suggests that the collection of *L. lactis* genomes is still not enough, indicating that there are more new genes to be discovered. Previous reports showed that bacterial genomes changed when they adapted to variable conditions, and that greater niche diversity corresponded to larger pangenomes (Konstantinidis and Tiedje, 2004). The genomes and metagenomes we collected are mainly from dairy niches where genome decay and redundancy are widely reported (Makarova et al., 2006; Goh et al., 2011; Ainsworth et al., 2013). Therefore, to better characterize the lactococcal pangenome, more lactococcal genomes from various niches need to be sequenced. Also, qualified genomes in our catalog, either MAGs or isolate genomes, only belong to two subspecies (i.e., *lactis* and *cremoris*). If more samples containing other subspecies are sequenced in the future, more diversity could be investigated. Furthermore, a limited number of *L. lactis* genomes recovered from non-food niches are available because of the low abundance of *L. lactis* in those environments. More genomes sampled from diverse environments, especially those uncultured strains and those from sources other than dairy products, can provide a better insight to the niche adaption of *L. lactis*, which can enhance the safety and flavor profiles of fermented dairy products.

In conclusion, the integration of isolate genomes and MAGs has enlarged the number of high-quality lactococcal genomes and allows for large-scale analysis of this taxon at both genome and gene levels. It will become a valuable resource for future genome-centric data mining and experimental validation. The deduced pangenome of *L. lactis* generated by the integrated genome catalog is still open, indicating that there is higher genetic diversity that needs to be discovered. What is more, we tried to combine MAGs and isolated genomes on pangenome analysis of an individual bacterial species. This idea of combining MAGs and isolate genomes for pan-genome analysis could also be applied to other species.

## Materials and methods

### Overview of the approach

Our approach to reconstructing bacterial genomes from the food metagenomes exploited metagenomic single-sample assembly, contig binning, and taxonomic annotation of genomes. Meanwhile, human microbiota MAGs and isolate genomes of *L. lactis* were downloaded from public databases. Quality control was then conducted on all the genomes. Genomes with quality at least comparable with the typical quality of isolate genome sequencing (completeness > 90% and contamination < 5%) were kept for consequent analysis.

### Genome collection

To retrieve isolate genomes, we surveyed NCBI database (*L. lactis* subsp. *lactis*:^1^ and *L. lactis* subsp. *Cremoris*:^2^) for *Lactococcus lactis* genomes publicly available as of Mar 2022 and downloaded 70 isolate genomes at complete or chromosome assembly level. MAGs (i.e., uncultured genomes) from the human gut were obtained from Pasolli et al. (SGB collection^3^) (Pasolli et al., 2019). This SGB collection contained 154,723 reconstructed microbial genomes with taxonomy annotation assembled from 9,428 samples of the human microbiome and we only used 29 genomes that were annotated as *L. lactis* and retrieved from samples collected from the human intestinal tract. A full list of all isolate genomes and MAGs used in this study can be found in Supplementary Table 2.

### Publicly available metagenomic datasets

We considered and curated 15 public datasets with shotgun sequencing metagenomic data taken from different fermented foods. In total, we put together 323 samples coming from cheese (*N* = 201), yogurt and dietary supplements (*N* = 16), soy sauce (*N* = 13), sausages (*N* = 16), wine (*N* = 16), Indonesia food (*N* = 3), and various fermented food (*N* = 58). The raw data were downloaded from the National Center Biotechnology Information (NCBI) and the metagenomics RAST (MG-RAST). For human metagenomes, we collected 3 publicly available metagenomic datasets corresponding to the human gut microbiome, totaling 1,123 metagenomes. In addition, we downloaded the corresponding 26 metagenomes where human microbiota MAGs mentioned above recovered (two were not available and one was a duplicate sample with project: PRJNA231909). Sequence names were simplified to contain only the Project accession and a unique Run accession. The metadata of all manually-curated metagenomes is available in Supplementary Table 1.

### Raw read filtering and quality control

We used KneadData^4^ to do trimming and quality control before assembly. This pipeline involved two primary steps. One was aligning raw reads back to the human genome reference (GRCh37/hg19) to filter out human contaminants. The second was doing stringent quality control using Trimmomatic (Bolger et al., 2014) (version 0.10.0; option “LEADING:3; TRAILING:3; MINLEN:50; SLIDINGWINDOW:4:20; HEADCROP:10; ILLUMINACLIP:/ NexteraPE-PE.fa:2:30:10”), in which the adapters were excised, reads were trimmed using a 4 bp sliding window with an average quality score threshold of Q20, and reads containing any ambiguous bases were removed.

### Taxonomic profiling of metagenomes based on short reads

We ran MetaPhlan3 (Beghini et al., 2021) (version 3.0.8; default parameters) to identify the species composition in each food and human metagenomic sample. That tool estimated the relative abundance of microbial species relying on ∼1.1 M unique clade-specific marker genes identified from ∼100,000 reference genomes (∼99,500 bacterial and archaeal and ∼500 eukaryotic). The output of each sample contained the species composition and relative abundance. Then we collapsed the outputs of all samples into a relative abundance matrix, where the columns were samples and the rows were species. Only when a relative abundance of a species in a sample was greater than 0.01%, we considered it existing in that sample. A taxonomic abundance table for all metagenomes was available in Supplementary Table 3.

### Metagenomic assembly and contig binning

We assembled clean reads into contigs using SPAdes (version 3.15.2; option “–meta”) which invoked the read corrector BayesHammer at first. Samples with only unpaired reads and samples that failed to be processed by SPAdes were assembled through MEGAHIT (Li et al., 2016) (version 1.2.9; default parameters). The sequence data of each sample were assembled individually.

The minimum length of contigs used for constructing bins was 1500 bp. Reads were mapped to contigs using Bowtie2 (Langdon, 2015) (version 2.4.4; default parameters) and the mapping output was then used for contig binning through MetaBAT2 (Kang et al., 2019) (version 2.12.1; option “-m 1500”). MetaBAT2 achieved the best performance among single-sample binning tools in the evaluation performed in the Metawrap (Uritskiy et al., 2018) paper. The procedure of binning through MetaBAT2 generated 2,086 metagenomic bins (or MAGs) in total.

### Quality control of metagenome-assembled genomes (MAGs) and isolate genomes

Constructed food microbiota MAGs were subjected to quality control to generate the final set of high-quality genomes. Two main measures were taken into account: (1) completeness; (2) contamination. Genome quality was estimated with CheckM (Parks et al., 2015) (version 1.1.3) using the ‘lineage_wf’ workflow. It estimated genome completeness and contamination by using collocated sets of genes that were ubiquitous and single-copy within a phylogenetic lineage. We selected medium-quality (MQ) genomes those having completeness > 50% and contamination < 5% resulting in a total of 894 bacterial genomes. Stricter quality control reduced the set of near-complete, high-quality (HQ) genomes to 497 with completeness > 90%.

Genome quality of all isolate genomes and human microbiota MAGs were also estimated with CheckM (Parks et al., 2015) to select genomes that passed the following criteria: > 90% completeness and < 5% contamination, to avoid possible biases coming from highly incomplete genomes. All 70 isolate genomes met the quality requirement and only 12 (out of 29) human microbiota MAGs survived. These qualified genomes were incorporated with high-quality food microbiota MAGs to be used for downstream species annotation.

### Taxonomic annotation of metagenome-assembled genomes (MAGs) and isolate genomes

A total of 579 high-quality genomes, including MAGs and isolates, were classified by two different tools: PhyloPhlAn (version 3.0.2; option “-n 1”) (Asnicar et al., 2020) and the Genome Taxonomy Database Toolkit (GTDB-Tk) (version 1.7.0; database release 202) using the “classify_wf” function and default parameters.

With the “phylophlan_metagenomic” module in PhyloPhlAn, we used the species-level genome bins (SGB) release of January 2019 (Pasolli et al., 2019) to assign each metagenome-assembled genome to its closest SGB. If the genome bin had a Mash distance < 5% from the reported SGB, we can consider that bin as part of it and assign the SGB’s taxonomic label. For GTDB-Tk, it assigned objective taxonomic classifications to bacterial and archaeal genomes based on phylogenetic analysis of a large number of genomes (Parks et al., 2018). The genome annotation results obtained by the two tools were consistent. The number of food microbiota MAGs annotated as *Lactococcus lactis* were eighty-one. Seventy isolate genomes and twelve human microbiota MAGs we collected were verified belonging *L. lactis*. Summaries of taxonomic labels were available in Supplementary Table 3.

### Removing redundancy of genome catalog and average nucleotide identity (ANI) calculation

We assumed that a pair of genomes were redundant if their average nucleotide identity (ANI) was at least 99.8% over the alignment between them that covered at least 50% of the shorter genome. dRep (Olm et al., 2017) (version 3.0.0; option “-pa 0.95 -sa 0.99 -nc 0.5 -cm larger”) was used for the dereplication of all 163 genomes (isolated genomes and MAGs recovered from food and human gut) by two-step cluster. First, genomes were divided into primary clusters using MASH at a 95% Mash ANI. Then, each primary cluster was used to form secondary clusters at the threshold of 99.8% ANI with at least 50% overlap between genomes.

For each group of redundant genomes, dRep chose the genome with the highest score as the representative of the group. A score was calculated for each genome on the basis of the following formula:

Score=A*Completeness-B*Contamination+C*

(Contamination*(strainheterogeneity/100))+D*log⁢(N50)

+E*log⁢(size)+F*(centrality-S⁢nia)

where A-F were arguments with default values of 1, 5, 1, 0.5, 0, and 1, respectively. Completeness, Contamination, and strain heterogeneity were calculated based on single-copy genes. N50 was a measure of how big the pieces were that made up the genome. Size was the total length of the genome. Centrality was a measure of how similar a genome was to all other genomes in their cluster. Finally, the number of non-redundant genomes belonging to *L. lactis* was 60.

In addition, to further investigate the within-species population diversity, we calculated pairwise distances for all conspecific genomes using fastANI (Jain et al., 2018) (version 1.32; default parameters). From these results, we generated a distance tree using the “complete” hierarchical clustering method.

### Pangenome, phylogenetic, and functional enrichment analysis

Protein-coding sequences (CDS) for each of the 163 genomes of *Lactococcus lactis* (MAGs and isolate genomes) were predicted and annotated with Prokka (version 1.14.6; default parameters) (Seemann, 2014). The annotated genomes were then processed with Roary (Page et al., 2015) (version 3.12.0; option “-i 80 -cd 95”) for the pangenome analysis and to generate the presence-absence binary matrix on the whole genes. Different sequencing alignment identity would result in different sizes of pangenome (Supplementary Figure 7). The identity used in this study was 80%, that is, genes with similarity greater than 80% were considered to be the same gene, while genes with similarity less than 80% were considered to be different genes. The core genes that occurred in nearly all genomes (> 95%) identified by Roary were then used for phylogenetic analysis. The program “anvi-display-pan” of Anvi’o pangenomics workflow (Delmont and Eren, 2018) provided interactive visualizations of pangenomes. To simplify visualizations of complex pangenomes, we removed singleton genes using the parameter “—min-occurrence 2”.

The maximum-likelihood tree was generated *de novo* using the core gene alignments produced by Roary. We used IQ-TREE (Minh et al., 2020) (version 2.2.0-beta; option “–m MFP -B 1000 –bnni”) to build a phylogenetic tree of the 60 non-redundant genomes. The best fit module was automatically selected by “ModelFinder” on the basis of the Bayesian information criterion (BIC) score. The UNREST+FO+R5 model was chosen for building the tree. The phylogenetic tree was visualized and annotated with Interactive Tree Of Life (iTOL) (Letunic and Bork, 2016) (version 3).

Functional annotation of all pangenome sequences was performed with EggNOG-mapper (Huerta-Cepas et al., 2016), and the sequence searches were performed using diamond. GO annotations were derived from the EggNOG-mapper results. Functional enrichment analysis was taken with clusterProfiler (Wu et al., 2021). We considered a function to be enriched if the p-value and q-value were below 0.05, which controlled the expected proportion of false positives at 0.05.

### Calculation of cross-sample dissimilarity

Using the relative abundance profiles of samples containing *L. lactis*, we created a distance matrix using Bray-Curtis Dissimilarity. Bray-curtis distance was a quantitative asymmetrical index whose advantage was ignoring the double zero problems.

## Data availability statement

The raw sequencing data for the food and human metagenomes are available in NCBI-SRA (https://www.ncbi.nlm.nih.gov/), and in MG-RAST under the Project mgp3362 (https://www.mg-rast.org/mgmain.html?mgpage=search&search=mgp3362). Human gut microbiota MAGs used to construct genome catalog have been retrieved from SGB collection: https://opendata.lifebit.ai/table/sgb. Bulk download for the 163 high-quality genomes of *L. lactis* is available at https://cgm.sjtu.edu.cn/Lactococcus_lactis-pangenome/genome_catalog_of_Lactococcus_lactis.zip. The pangenome sequences, PAV tables of each pangenome, and functional information are available at https://github.com/skye-zhai/analysis-pipeline. All codes used in this study are publicly available at https://github.com/skye-zhai/analysis-pipeline.

## Author contributions

CW and YZ conceived the study and wrote the manuscript. YZ implemented the analysis pipeline, collected the genomic sequence data, and analyzed the data. Both authors reviewed the manuscript.

## Footnotes

1
https://www.ncbi.nlm.nih.gov/genome/?term=lactococcus+lactis

2
https://www.ncbi.nlm.nih.gov/genome/?term=lactococcus+cremoris

3
https://opendata.lifebit.ai/table/sgb

4
https://github.com/biobakery/kneaddata

## Supplementary material

Supplementary Table 1 Food and Human metagenomic datasets.

Supplementary Table 2 Metadata of isolate genomes and quality control results of metagenome-assembled genomes (MAGs) and isolate genomes.

Supplementary Table 3 Merged species abundance table of all 323 food metagenomes; Merged species abundance table of all *Lactococcus lactis* positive metagenomes (including 217 food and 106 human gut metagenomes).

## References

[B1] Ainsworth S. Zomer A. de Jager V. Bottacini F. van Hijum S. A. Mahony J. (2013). Complete Genome of Lactococcus lactis subsp. cremoris UC509.9, Host for a Model Lactococcal P335 Bacteriophage. *Genome Announc.* 1:e00119–12. 23405300 10.1128/genomeA.00119-12 PMC3569286

[B2] Almeida A. Nayfach S. Boland M. Strozzi F. Beracochea M. Shi Z. J. (2021). A unified catalog of 204,938 reference genomes from the human gut microbiome. *Nat. Biotechnol.* 39 105–114. 10.1038/s41587-020-0603-3 32690973 PMC7801254

[B3] Asnicar F. Thomas A. M. Beghini F. Mengoni C. Manara S. Manghi P. (2020). Precise phylogenetic analysis of microbial isolates and genomes from metagenomes using PhyloPhlAn 3.0. *Nat. Commun.* 11:2500. 10.1038/s41467-020-16366-7 32427907 PMC7237447

[B4] Beghini F. McIver L. J. Blanco-Míguez A. Dubois L. Asnicar F. Maharjan S. (2021). Integrating taxonomic, functional, and strain-level profiling of diverse microbial communities with bioBakery 3. *eLife* 10:e65088. 10.7554/eLife.65088 33944776 PMC8096432

[B5] Bolger A. M. Lohse M. Usadel B. (2014). Trimmomatic: A flexible trimmer for Illumina sequence data. *Bioinformatics* 30 2114–2120. 24695404 10.1093/bioinformatics/btu170 PMC4103590

[B6] Bosi E. Fani R. Fondi M. (2015). Defining orthologs and pangenome size metrics. *Methods Mol. Biol.* 1231 191–202. 10.1007/978-1-4939-1720-4_13 25343867

[B7] Breitwieser F. P. Salzberg S. L. (2020). Pavian: Interactive analysis of metagenomics data for microbiome studies and pathogen identification. *Bioinformatics* 36 1303–1304. 10.1093/bioinformatics/btz715 31553437 PMC8215911

[B8] Cavanagh D. Fitzgerald G. F. McAuliffe O. (2015b). From field to fermentation: The origins of Lactococcus lactis and its domestication to the dairy environment. *Food Microbiol.* 47 45–61. 10.1016/j.fm.2014.11.001 25583337

[B9] Cavanagh D. Casey A. Altermann E. Cotter P. D. Fitzgerald G. F. McAuliffe O. (2015a). Evaluation of Lactococcus lactis Isolates from Nondairy Sources with Potential Dairy Applications Reveals Extensive Phenotype-Genotype Disparity and Implications for a Revised Species. *Appl. Environ. Microbiol.* 81 3961–3972. 10.1128/AEM.04092-14 25841018 PMC4524136

[B10] Chun B. H. Kim K. H. Jeon H. H. Lee S. H. Jeon C. O. (2017). Pan-genomic and transcriptomic analyses of Leuconostoc mesenteroides provide insights into its genomic and metabolic features and roles in kimchi fermentation. *Sci. Rep.* 7:11504. 10.1038/s41598-017-12016-z 28912444 PMC5599536

[B11] Delmont T. O. Eren A. M. (2018). Linking pangenomes and metagenomes: The Prochlorococcus metapangenome. *Peerj* 6:e4320. 10.7717/peerj.4320 29423345 PMC5804319

[B12] Dowdell P. Chankhamhaengdecha S. Panbangred W. Janvilisri T. Aroonnual A. (2020). Probiotic Activity of Enterococcus faecium and Lactococcus lactis Isolated from Thai Fermented Sausages and Their Protective Effect Against Clostridium difficile. *Probiotics Antimicrob. Proteins* 12 641–648. 10.1007/s12602-019-09536-7 30888623 PMC7306037

[B13] Dutilh B. E. Huynen M. A. Bruno W. J. Snel B. (2004). The consistent phylogenetic signal in genome trees revealed by reducing the impact of noise. *J. Mol. Evol.* 58 527–539. 10.1007/s00239-003-2575-6 15170256

[B14] Food and Drug Administration [FDA] (2010). *Generally Recognised as Safe (GRAS) Notifications.* Silver Spring: Food and Drug Administration.

[B15] Gao Y. Lu Y. Teng K. L. Chen M. L. Zheng H. J. Zhu Y. Q. (2011). Complete genome sequence of Lactococcus lactis subsp. lactis CV56, a probiotic strain isolated from the vaginas of healthy women. *J. Bacteriol.* 193 2886–2887. 10.1128/JB.00358-11 21460077 PMC3133120

[B16] Goh Y. J. Goin C. O’flaherty S. Altermann E. Hutkins R. (2011). Specialized adaptation of a lactic acid bacterium to the milk environment: The comparative genomics of Streptococcus thermophilus LMD-9. *Microb. Cell Fact.* 10:S22. 10.1186/1475-2859-10-S1-S22 21995282 PMC3231929

[B17] Huerta-Cepas J. Szklarczyk D. Forslund K. Cook H. Heller D. Walter M. C. (2016). eggNOG 4.5: A hierarchical orthology framework with improved functional annotations for eukaryotic, prokaryotic and viral sequences. *Nucleic Acids Res.* 44 D286–D293. 10.1093/nar/gkv1248 26582926 PMC4702882

[B18] Jain C. Rodriguez-R L. M. Phillippy A. M. Konstantinidis K. T. Aluru S. (2018). High throughput ANI analysis of 90K prokaryotic genomes reveals clear species boundaries. *Nat. Commun.* 9:5114. 10.1038/s41467-018-07641-9 PMC6269478 30504855

[B19] Jung J. Y. Lee S. H. Kim J. M. Park M. S. Bae J. W. Hahn Y. (2011). Metagenomic analysis of kimchi, a traditional Korean fermented food. *Appl. Environ. Microbiol.* 77 2264–2274. 10.1128/AEM.02157-10 21317261 PMC3067442

[B20] Kang D. D. Li F. Kirton E. Thomas A. Egan R. An H. (2019). MetaBAT 2: An adaptive binning algorithm for robust and efficient genome reconstruction from metagenome assemblies. *PeerJ* 7:e7359. 10.7717/peerj.7359 31388474 PMC6662567

[B21] Kato H. Shiwa Y. Oshima K. Machii M. Araya-Kojima T. Zendo T. (2012). Complete Genome Sequence of Lactococcus lactis IO-1, a Lactic Acid Bacterium That Utilizes Xylose and Produces High Levels of L-Lactic Acid. *J. Bacteriol.* 194 2102–2103. 10.1128/JB.00074-12 22461545 PMC3318493

[B22] Kelleher P. Bottacini F. Mahony J. Kilcawley K. N. van Sinderen D. (2017). Comparative and functional genomics of the Lactococcus lactis taxon; insights into evolution and niche adaptation. *BMC Genomics* 18:267. 10.1186/s12864-017-3650-5 28356072 PMC5372332

[B23] Kim C. Y. Lee M. Yang S. Kim K. Yong D. Kim H. R. (2021). Human reference gut microbiome catalog including newly assembled genomes from under-represented Asian metagenomes. *Genome Med.* 13:134. 10.1186/s13073-021-00950-7 34446072 PMC8394144

[B24] Konstantinidis K. T. Tiedje J. M. (2004). Trends between gene content and genome size in prokaryotic species with larger genomes. *Proc. Natl. Acad. Sci. U.S.A.* 101 3160–3165. 10.1073/pnas.0308653100 14973198 PMC365760

[B25] Langdon W. B. (2015). Performance of genetic programming optimised Bowtie2 on genome comparison and analytic testing (GCAT) benchmarks. *BioData Min.* 8:1. 10.1186/s13040-014-0034-0 25621011 PMC4304608

[B26] Laroute V. Tormo H. Couderc C. Mercier-Bonin M. Le Bourgeois P. (2017). From Genome to Phenotype: An Integrative Approach to Evaluate the Biodiversity of Lactococcus lactis. *Microorganisms* 5:27. 10.3390/microorganisms5020027 28534821 PMC5488098

[B27] Letunic I. Bork P. (2016). Interactive tree of life (iTOL) v3: An online tool for the display and annotation of phylogenetic and other trees. *Nucleic Acids Res.* 44 W242–W245. 10.1093/nar/gkw290 27095192 PMC4987883

[B28] Li D. Luo R. Liu C. M. Leung C. M. Ting H. F. Sadakane K. (2016). MEGAHIT v1.0: A fast and scalable metagenome assembler driven by advanced methodologies and community practices. *Methods* 102 3–11. 10.1016/j.ymeth.2016.02.020 27012178

[B29] Makarova K. Slesarev A. Wolf Y. Sorokin A. Mirkin B. Koonin E. (2006). Comparative genomics of the lactic acid bacteria. *Proc. Natl. Acad. Sci. U.S.A.* 103 15611–15616. 17030793 10.1073/pnas.0607117103 PMC1622870

[B30] Mataragas M. (2020). Investigation of genomic characteristics and carbohydrates’ metabolic activity of Lactococcus lactis subsp. lactis during ripening of a Swiss-type cheese. *Food Microbiol.* 87:103392. 10.1016/j.fm.2019.103392 31948633

[B31] Meziti A. Rodriguez-R L. M. Hatt J. K. Peña-Gonzalez A. Levy K. Konstantinidis K. T. (2021). The Reliability of Metagenome-Assembled Genomes (MAGs) in Representing Natural Populations: Insights from Comparing MAGs against Isolate Genomes Derived from the Same Fecal Sample. *Appl. Environ. Microbiol.* 87:e02593–20. 10.1128/AEM.02593-20 33452027 PMC8105024

[B32] Minh B. Q. Schmidt H. A. Chernomor O. Schrempf D. Woodhams M. D. von Haeseler A. (2020). IQ-TREE 2: New Models and Efficient Methods for Phylogenetic Inference in the Genomic Era. *Mol. Biol. Evol.* 37 1530–1534. 32011700 10.1093/molbev/msaa015 PMC7182206

[B33] Nayfach S. Roux S. Seshadri R. Udwary D. Varghese N. Schulz F. (2021). A genomic catalog of Earth’s microbiomes. *Nat. Biotechnol.* 39 499–509. 33169036 10.1038/s41587-020-0718-6 PMC8041624

[B34] Neves A. R. Pool W. A. Kok J. Kuipers O. P. Santos H. (2005). Overview on sugar metabolism and its control in Lactococcus lactis - the input from in vivo NMR. *FEMS Microbiol. Rev.* 29 531–554. 10.1016/j.femsre.2005.04.005 15939503

[B35] Olm M. R. Brown C. Brooks B. (2017). Banfield J F dRep: A tool for fast and accurate genomic comparisons that enables improved genome recovery from metagenomes through de-replication. *ISME J.* 11 2864–2868. 10.1038/ismej.2017.126 28742071 PMC5702732

[B36] Page A. J. Cummins C. A. Hunt M. Wong V. K. Reuter S. Holden M. T. (2015). Roary: Rapid large-scale prokaryote pan genome analysis. *Bioinformatics* 31 3691–3693. 10.1093/bioinformatics/btv421 26198102 PMC4817141

[B37] Parks D. H. Chuvochina M. Waite D. W. Rinke C. Skarshewski A. Chaumeil P. A. (2018). A standardized bacterial taxonomy based on genome phylogeny substantially revises the tree of life. *Nat. Biotechnol.* 36 996–1004. 10.1038/nbt.4229 30148503

[B38] Parks D. H. Imelfort M. Skennerton C. T. Hugenholtz P. Tyson G. W. (2015). CheckM: Assessing the quality of microbial genomes recovered from isolates, single cells, and metagenomes. *Genome Res.* 25 1043–1055. 10.1101/gr.186072.114 25977477 PMC4484387

[B39] Pasolli E. Asnicar F. Manara S. Zolfo M. Karcher N. Armanini F. (2019). Extensive Unexplored Human Microbiome Diversity Revealed by Over 150,000 Genomes from Metagenomes Spanning Age, Geography, and Lifestyle. *Cell* 176 649–662.e20. 10.1016/j.cell.2019.01.001 30661755 PMC6349461

[B40] Pasolli E. De Filippis F. Mauriello I. E. Cumbo F. Walsh A. M. Leech J. (2020). Large-scale genome-wide analysis links lactic acid bacteria from food with the gut microbiome. *Nat. Commun.* 11:2610. 10.1038/s41467-020-16438-8 PMC7248083 32451391

[B41] Passerini D. Beltramo C. Coddeville M. Quentin Y. Ritzenthaler P. Daveran-Mingot M. L. (2010). Genes but not genomes reveal bacterial domestication of Lactococcus lactis. *PLoS One* 5:e15306. 10.1371/journal.pone.0015306 21179431 PMC3003715

[B42] Perez T. Balcázar J. L. Peix A. Valverde A. Velázquez E. de Blas I. (2011). Lactococcus lactis subsp. tructae subsp. nov. isolated from the intestinal mucus of brown trout (Salmo trutta) and rainbow trout (Oncorhynchus mykiss). *Int. J. Syst. Evol. Microbiol.* 61 1894–1898. 10.1099/ijs.0.023945-0 20833888

[B43] Seemann T. (2014). Prokka: Rapid prokaryotic genome annotation. *Bioinformatics* 30 2068–2069. 24642063 10.1093/bioinformatics/btu153

[B44] Shaiber A. Willis A. D. Delmont T. O. Roux S. Chen L. X. (2020). Functional and genetic markers of niche partitioning among enigmatic members of the human oral microbiome. *Genome Biol.* 21:292. 10.1186/s13059-020-02195-w 33323122 PMC7739484

[B45] Tettelin H. Masignani V. Cieslewicz M. J. Donati C. Medini D. Ward N. L. (2005). Genome analysis of multiple pathogenic isolates of Streptococcus agalactiae: Implications for the microbial “pan-genome”. *Proc. Natl. Acad. Sci. U.S.A.* 102 13950–13955. 10.1073/pnas.0506758102 16172379 PMC1216834

[B46] Todorov S. D. Botes M. Danova S. T. Dicks L. M. (2007). Probiotic properties of Lactococcus lactis ssp. lactis HV219, isolated from human vaginal secretions. *J. Appl. Microbiol.* 103 629–639. 10.1111/j.1365-2672.2007.03290.x 17714396

[B47] Uritskiy G. V. DiRuggiero J. Taylor J. (2018). MetaWRAP-a flexible pipeline for genome-resolved metagenomic data analysis. *Microbiome* 6:158. 10.1186/s40168-018-0541-1 30219103 PMC6138922

[B48] Wu T. Hu E. Xu S. Chen M. Guo P. Dai Z. (2021). clusterProfiler 4.0: A universal enrichment tool for interpreting omics data. *Innovation* 2:100141. 10.1016/j.xinn.2021.100141 34557778 PMC8454663

[B49] Xu J. (2006). Microbial ecology in the age of genomics and metagenomics: Concepts, tools, and recent advances. *Mol. Ecol.* 15 1713–1731. 10.1111/j.1365-294X.2006.02882.x 16689892

