# Supplementary material for: Open pangenome of Lactococcus lactis generated by a combination of metagenome-assembled genomes and isolate genomes
Source: Front Microbiol. 2022 Aug 23;13:948138. doi: 10.3389/fmicb.2022.948138 (PMC9445621; doi:10.3389/fmicb.2022.948138)
Supplement: Supplementary file 1 [file Data_Sheet_1.PDF]

Supplementary Materials for

Open pangenome of *Lactococcus lactis* generated by a combination of metagenome-assembled genomes (MAGs) and isolate genomes

Yiting Zhai, Chaochun Wei

Supplementary Figures

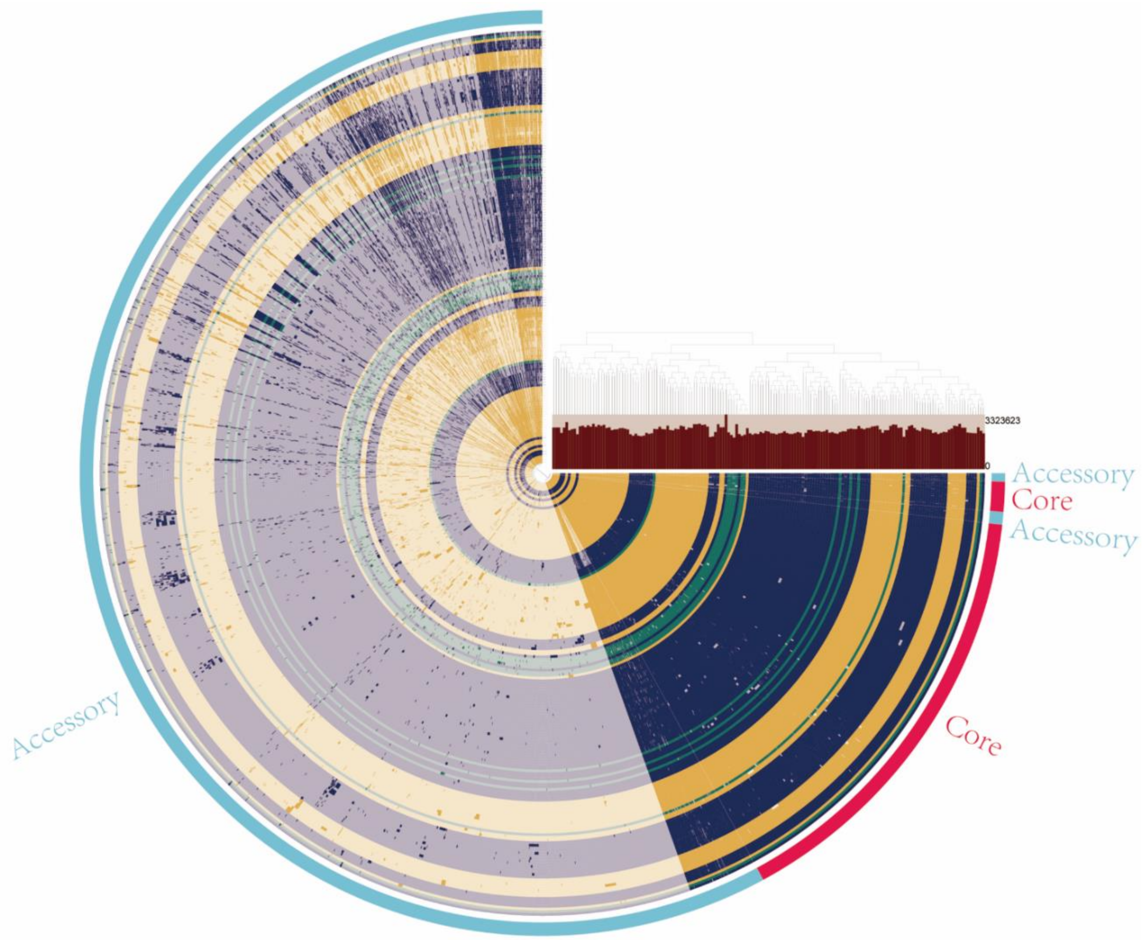

Supplementary Figure 1. The presence and absence of non-singleton genes in pangenome of *L. lactis*. The dendrogram in the center of the figure organizes the 6,741 non-singleton genes that occur in more than one genome according to their frequency of occurrence in the 163 genomes. The 163 inner layers correspond to the 163 genomes, where MAGs that are associated with human and food are shown in blue and green, isolate genomes are marked in yellow. The data points in the concentric layers represent the presence of a gene in a given genome, and the outermost circular layer highlights groups of genes that correspond to the core/accessory genes. Genomes in this figure are ordered according to their phylogenomic organization which is shown at the top-right corner. The top horizontal layer underneath the phylogenomic tree displays genome length for each genome.

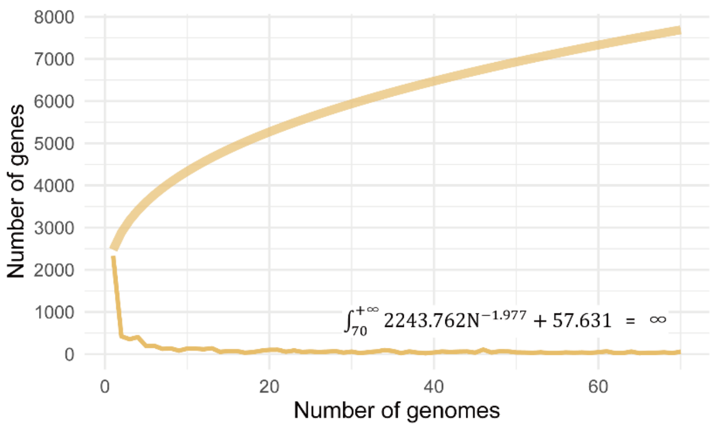

Supplementary Figure 2. Pangenome generated only by isolate genomes. The growth curve represents the number of total genes in pangenome, and the decreasing curve represents the number of new genes. Estimating the size of new genes based on a power function ($n=kN^r+a$), where n represents the number of additional new genes detected for each additional genome sample, N represents the number of genome samples, and k, r and a are three parameters of this model. The points used for fitting are the mean of 10 random sampling. The fitting result is k = 2243.762 (2153.396, 2334.182); r = -1.977 (-2.196, -1.796); a = 57.631 (45.435, 69.658) (R^2 = 0.975), and the integral is infinite: $\int_{70}^{+\infty} 2243.762N^{-1.977} + 57.631 = \infty$. The values in brackets are 95% confidence intervals. As for fitting of total number of genes, the deduced mathematical function is $y=1429.533x^{0.362}+1047.432$, $R^2=0.998$. With the increasing number of genomes, the size of the pangenome continues to grow and does not reach a plateau stage, indicating an open pangenome.

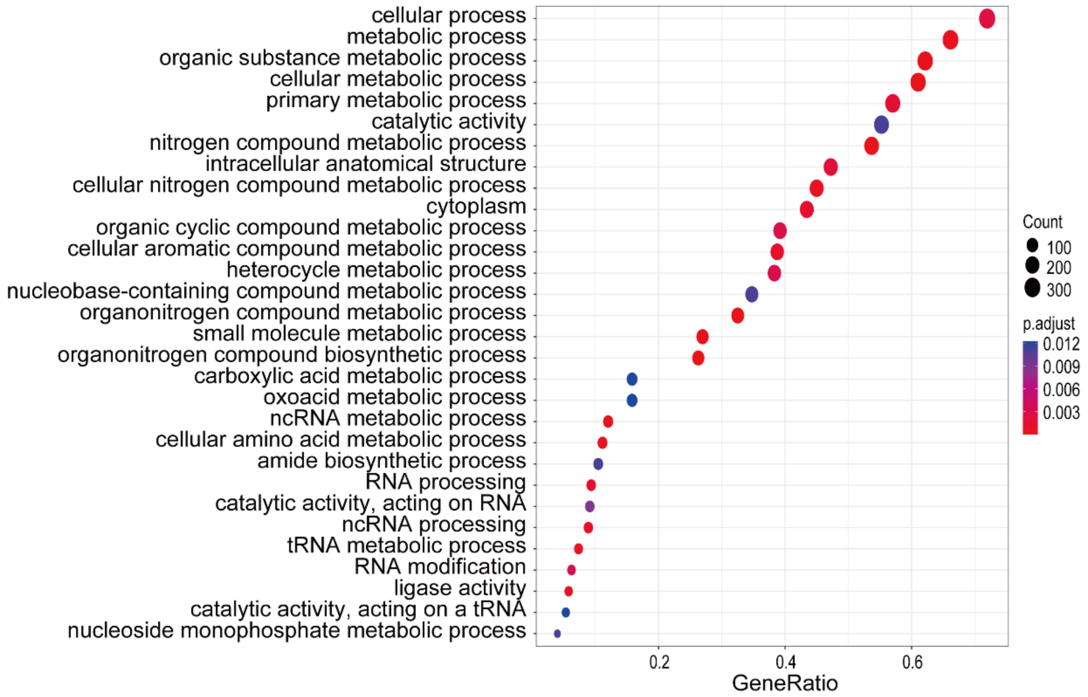

Supplementary Figure 3. GO functional enrichment analysis of core genes. The y-axis represents significantly enriched GO modules in the selected gene set, and the x-axis shows the occurrence proportion of each GO module within the gene set. The bubble color corresponds to the p. adjust (BH-corrected P) value. Only the top 30 GO modules with the lowest p. adjust values were shown.

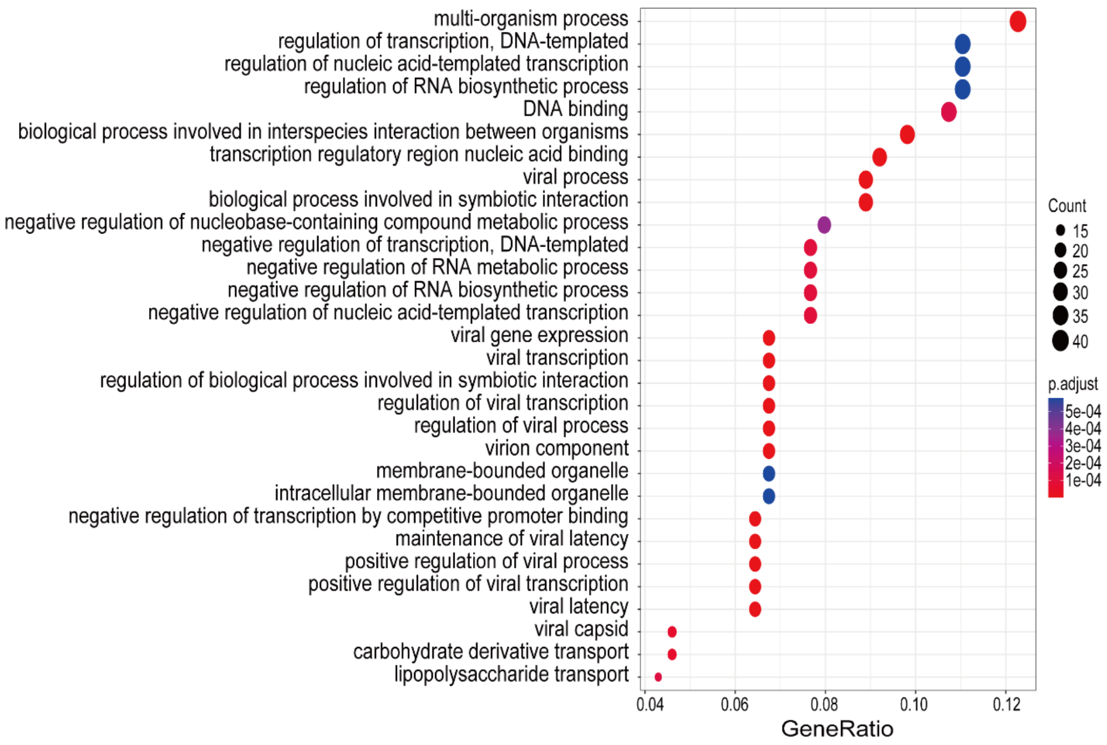

Supplementary Figure 4. GO functional enrichment analysis of accessory genes. The illustration of this figure is the same as that of Supplementary Figure 3.

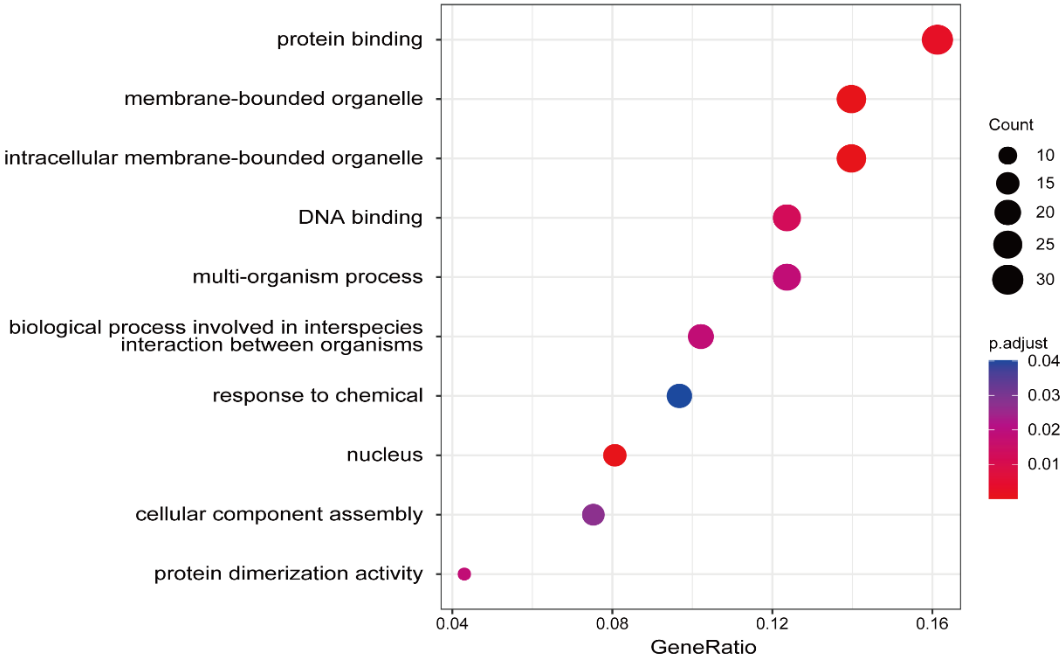

Supplementary Figure 5. GO functional enrichment analysis of singleton genes. The illustration of this figure is the same as that of Supplementary Figure 3.

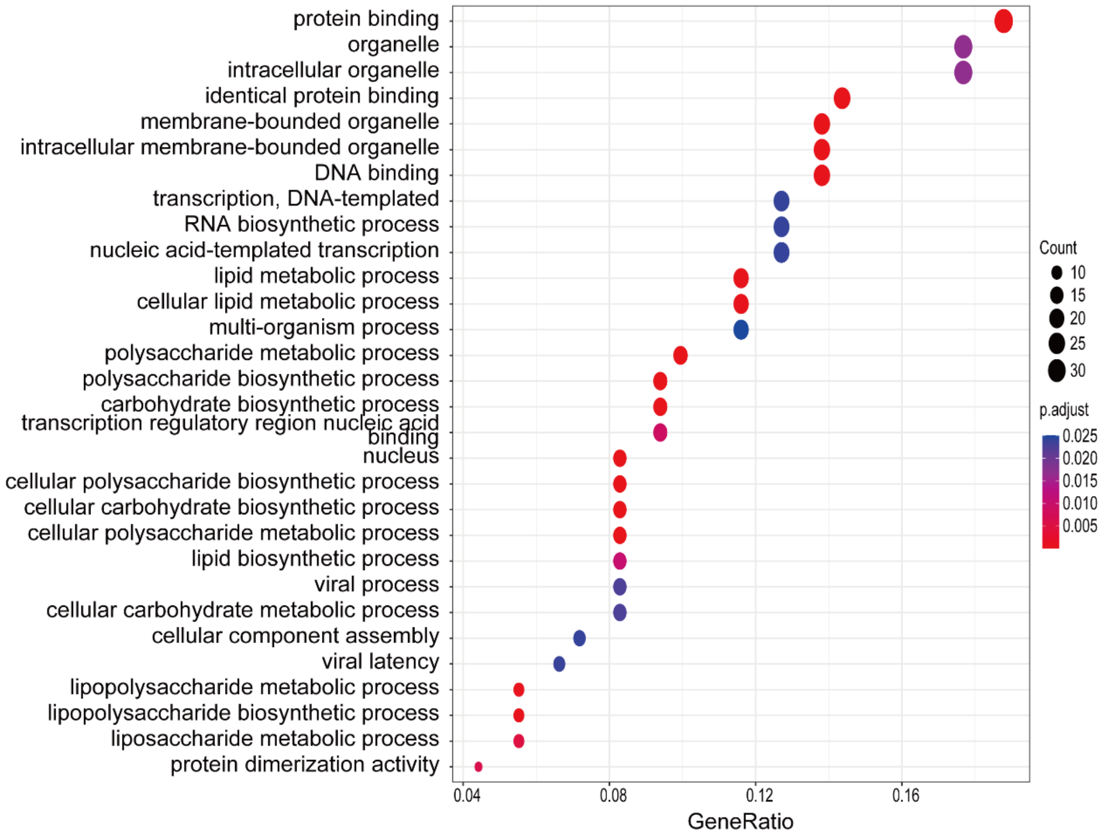

Supplementary Figure 6. GO functional modules enriched in MAGs. The illustration of this figure is the same as that of Supplementary Figure 3.
